# A Comparison Analysis of Four Different Drying Treatments on the Volatile Organic Compounds of Gardenia Flowers

**DOI:** 10.3390/molecules29184300

**Published:** 2024-09-11

**Authors:** Jiangli Peng, Wen Ai, Xinyi Yin, Dan Huang, Shunxiang Li

**Affiliations:** 1 Hunan Engineering Technology Research Center for Bioactive Substance Discovery of Chinese Medicine, School of Pharmacy, Hunan University of Chinese Medicine, Changsha 410208, China; janepeng@hnucm.edu.cn (J.P.); aiwen@stu.hnucm.edu.cn (W.A.); yxy@stu.hnucm.edu.cn (X.Y.); 2 Hunan Province Sino-US International Joint Research Center for Therapeutic Drugs of Senile Degenerative Diseases, Changsha 410208, China; 3 State Key Laboratory of Chinese Medicine Powder and Medicine Innovation in Hunan (Incubation), Science and Technology Innovation Center, Hunan University of Chinese Medicine, Changsha 410208, China

**Keywords:** gardenia flower, volatile organic compounds, drying method, HS-GC–IMS, PCA, CA, PLS-DA

## Abstract

The gardenia flower not only has extremely high ornamental value but also is an important source of natural food and spices, with a wide range of uses. To support the development of gardenia flower products, this study used headspace gas chromatography–ion mobility spectrometry (HS-GC–IMS) technology to compare and analyze the volatile organic compounds (VOCs) of fresh gardenia flower and those after using four different drying methods (vacuum freeze-drying (VFD), microwave drying (MD), hot-air drying (HAD), and vacuum drying (VD)). The results show that, in terms of shape, the VFD sample is almost identical to fresh gardenia flower, while the HAD, MD, and VD samples show significant changes in appearance with clear wrinkling; a total of 59 volatile organic compounds were detected in the gardenia flower, including 13 terpenes, 18 aldehydes, 4 esters, 8 ketones, 15 alcohols, and 1 sulfide. Principal component analysis (PCA), cluster analysis (CA), and partial least-squares regression analysis (PLS-DA) were performed on the obtained data, and the research found that different drying methods impact the VOCs of the gardenia flower. VFD or MD may be the most effective alternative to traditional sun-drying methods. Considering its drying efficiency and production cost, MD has the widest market prospects.

## 1. Introduction

The gardenia flower is the flower of *Gardenia jasminoides* Ellis. It has extremely high ornamental value but also is an important source of natural food and spices, with a wide range of uses. It is primarily produced in Zhejiang, Fujian, Jiangxi, Hunan, and Guangdong in China and is also distributed in Africa, Oceania, and Southeast Asia [1,2,3]. In South China, gardenia flowers are frequently incorporated into culinary dishes, including soups, stir-fries, and cold salads. Modern research has revealed that the gardenia flower is rich in vitamins, amino acids, trace elements, and various bioactive compounds, such as flavonoids, iridoids, triterpenes, and phenolic acids [4]. Phenols and iridoids have been identified as the primary functional components of the gardenia flower. Pharmacological research has primarily confirmed its anti-aging effects [5], anti-hyperlipidemia effects [6], anti-inflammatory effects [7,8], anti-anxiety effects [3], anti-depressant effects [9], and anti-bacterial activity [10]. It can also be utilized not only for food and medicine but also as a source for extracting aromatic compounds, which can be used as a blending agent in various floral cosmetics and soap essences.

The flavor in aromatic plant materials results from the interaction of taste and smell characteristics. Among these, the composition and content of volatile organic compounds (VOCs) play a significant role in the flavor characteristics of the final product [11].

Fresh gardenia flowers, upon harvest, are prone to browning due to their detachment from the growing environment and exposure to high temperatures, resulting in a loss of product quality and aroma post-harvest. Therefore, fresh gardenia flowers are commonly processed to preserve their aroma and extend their shelf life. Different drying techniques may affect the VOCs [12].

At present, the commonly used drying methods for food include vacuum freeze-drying (VFD), microwave drying (MD), hot-air drying (HAD), and vacuum drying (VD), among other technologies [13]. The drying process can eliminate up to 85%–95% of the moisture content in food, thereby inhibiting microbial spoilage and reducing water-mediated degradation reactions [14]. During the drying process, the Maillard reaction, protein degradation, and lipid oxidation give food unique flavor characteristics [15]. This flavor change also affects product quality, and a favorable flavor is an important factor in consumer purchase decisions. Therefore, understanding the influence of drying methods on the flavor of gardenia flowers is valuable. Due to varying energy transfer, different drying methods result in different drying times and product quality [16]. Choosing the appropriate drying process has a significant impact on the physicochemical and sensory quality of processed foods [17]. Suo et al. [14] found that cherry blossoms treated with VFD retained higher levels of bioactive compounds, antioxidant activity, alpha glucosidase inhibitory ability, and better flavor and taste characteristics compared to those treated with HAD, infrared hot-air drying (IHAD), catalytic infrared drying (CID), relative humidity drying (RHD), VD, and MD, but VFD required the longest drying time. Baibuch et al. [18] found that both VFD and HAD can maintain a relatively stable total phenolic content and antioxidant activity of rose petals, but VFD is more effective in preserving the red color of roses. Ma et al. [19] found that VFD of truffles is better than thermal drying because VFD has less effect on the lipid composition of truffles, keeping them similar to the lipid composition of fresh truffles.

Common methods for detecting VOCs include gas chromatography–mass spectrometry (GC-MS), gas chromatography × gas chromatography time-of-flight mass spectrometry (GC × GC TOF-MS), and gas chromatography–ion mobility spectrometry (GC–IMS) [20]. Gas chromatography–ion mobility spectrometry (GC–IMS) is a recently emerging technique for VOC analysis that combines gas chromatography with ion mobility spectrometry for rapid VOC detection [21]. In addition to accurately reflecting the VOCs in the sample, this method is faster, more accurate, and cheaper than traditional GC-MS analysis [21,22]. At the same time, using headspace injection to analyze low-temperature VOCs does not require enrichment or concentration, maximizing flavor preservation and ensuring more realistic results.

This study primarily investigates the differences in volatile organic compounds (VOCs) in fresh gardenia flowers after treatment with four different drying methods (vacuum freeze-drying, microwave drying, conventional drying, and vacuum drying). Utilizing HS-GC–IMS technology and chemometric methods, this study visually reveals significant differences in the content of volatile organic compounds among the four groups of materials. This experiment aims to verify the impact of different drying methods on the composition of volatile compounds and, through component analysis, select the most valuable drying method, providing a scientific basis and technical support for the further development of gardenia products.

The results of this study not only contribute to optimizing the drying process of gardenia flowers, enhancing their commercial value as food additives or flavorings, but also provide a new perspective on the application of natural flavors in the food industry. Through precise chemical analysis and data interpretation, this study opens up new possibilities for the processing and utilization of gardenia flowers, promoting technological advancement in related fields.

## 2. Results

### 2.1. The Influence of Different Drying Methods on the Appearance and Characteristics of Gardenia Flowers

**Figure 1:**
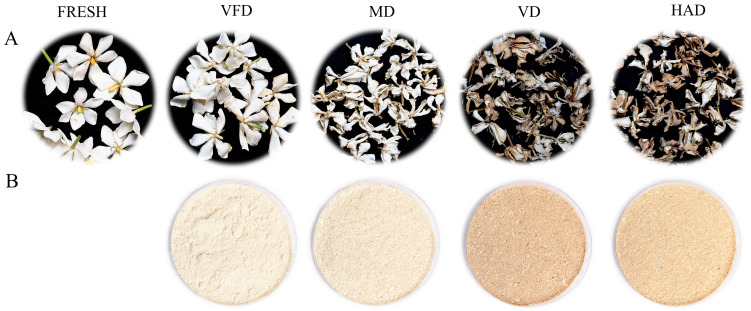
Photos of gardenia flowers (**A**) and powder (**B**) under different drying methods.

Figure 1 shows the appearance of gardenia flowers before and after drying using different methods. In terms of shape, the VFD sample is almost identical to fresh gardenia flowers, while the HAD, MD, and VD samples show significant changes in appearance, with clear wrinkling. Due to different drying conditions, the color of the gardenia flowers also changes significantly. VFD is closest in color to fresh gardenia flowers, and the MD gardenia flowers exhibit slight browning. In contrast, the HAD and VD samples show a higher browning index, the darkest color, and the greatest changes in appearance. Different drying conditions lead to varying sensory impacts. The distinct methods of drying cause significant changes in volatile compounds, with an increase in the content of aldehydes and ketones after drying the gardenia flowers. The production of Maillard reaction products (MRP) induced by drying also results in changes in the color parameters, while inevitably adversely affecting the nutritional characteristics [23].

### 2.2. GC–IMS Analysis of VOCs in the Five Groups of Gardenia Flowers

#### 2.2.1. Comparison of VOCs in the Five Groups of Gardenia Flowers

**Figure 2:**
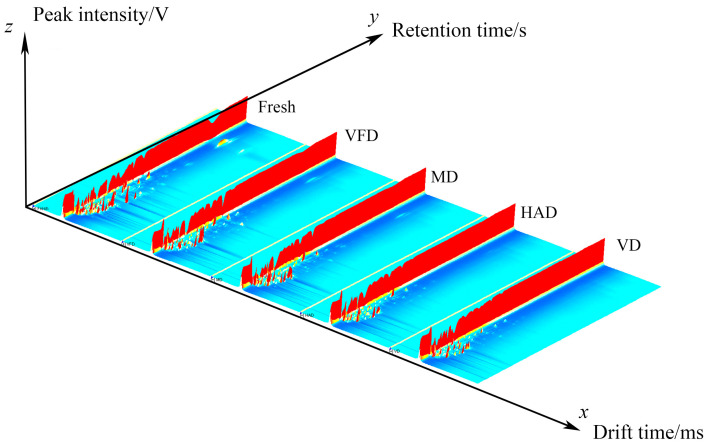
The 3D spectrum of the volatile organic compounds (VOCs) of five groups of gardenia flowers.

Figure 2 shows the three-dimensional spectra of the VOCs in gardenia flowers generated by different drying methods using the Reporter plugin program in GC–IMS instrument analysis software (from G.A.S., Dortmund, Germany, version 2.0.0). The x, y, and z axes represent the migration time, gas chromatography retention time, and peak intensity, respectively. There is a peak for each volatile component, and the height of each red peak represents how strong the signal is. Higher red protrusions indicate stronger signals and higher component contents. Weak signals and lower component contents are indicated by lower red protrusions. It can be seen from Figure 2 that drying gardenia flowers using different methods can result in different levels of VOCs.

Additionally, by combining the two-dimensional top view and differential spectra, we further investigated the GC–IMS.

**Figure 3:**
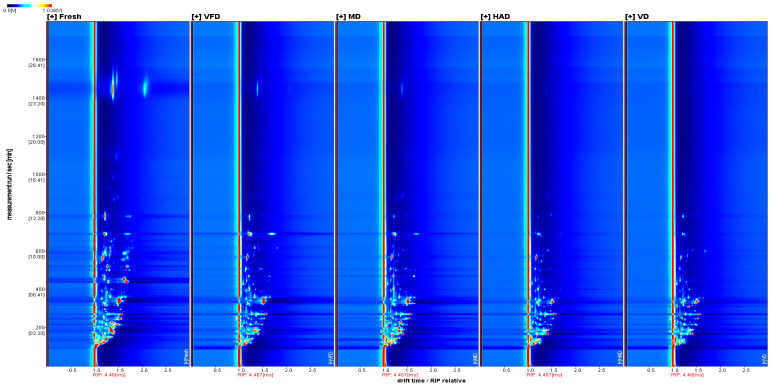
The 2D spectrum of the VOCs of five groups of gardenia flowers.

Figure 3 shows a two-dimensional top view of VOCs in gardenia flowers generated by different drying methods, with the horizontal axis representing the relative migration time and the vertical axis representing the retention time. The red vertical line represents the peak of reactive ions, while each bright spot represents a volatile component. It is possible to determine the content of VOCs by the color and area of the bright spot. The darker the color and the larger the area of the bright spot, the higher the content of the component. In terms of content, red is higher while blue is lower. From Figure 3, the differences in the VOCs in the gardenia flower samples treated with different drying methods can also be visually compared.

**Figure 4:**
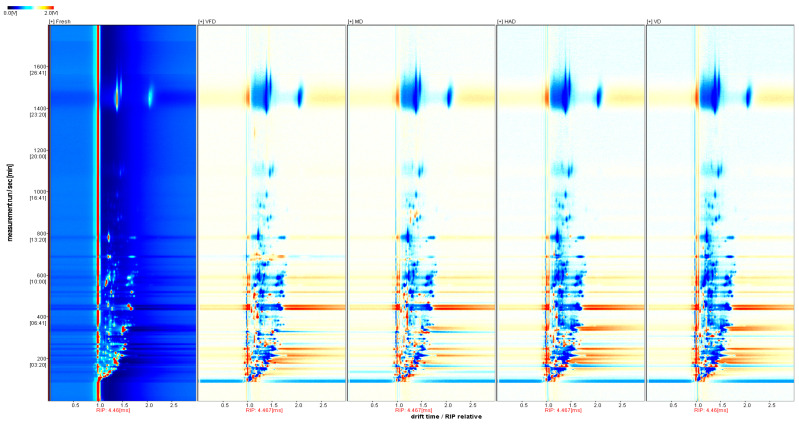
Analysis of the spectral differences between the fresh group and the other four groups of gardenia flowers.

In Figure 4, the spectrum of the fresh sample is shown as a reference, and those of the other samples are subtracted from this reference in order to obtain a comparison of the differences between the samples. When the volatile organic compound content in the target sample and reference is identical, the subtracted background is white, with red indicating that the substance concentration in the target sample is higher than that of the reference, and with blue indicating that it is lower. By analyzing the differential spectra, we can distinctly observe the differences between various drying methods for treating the gardenia samples. Compared to the fresh gardenia flowers, the signal changes at the drift time of 1.0–1.8 are the most pronounced, indicating that the content of these volatile compounds changed when the gardenia flower samples were dried under different conditions.

#### 2.2.2. Qualitative Analysis of VOCs in Groups of Gardenia Flowers by GC–IMS

**Figure 5:**
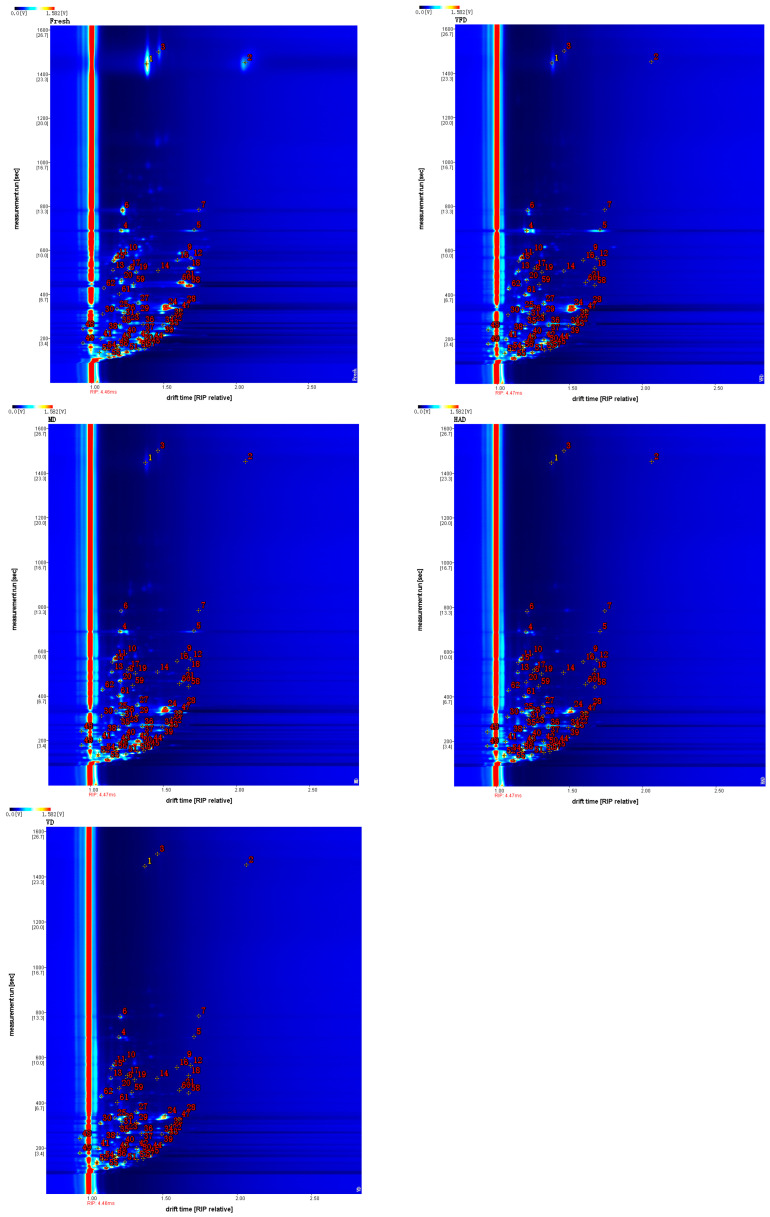
Qualitative spectrum of VOCs in the five groups of gardenia flowers based on gas chromatography–ion mobility spectrometry (GC–IMS).

To compare the relative differences in VOCs across five types of gardenia flowers, the data were processed using a VOCal plugin to obtain ion migration spectra for the five gardenia flower samples (Figure 5). Different points in this figure represent different VOCs; a darker color indicates higher content, while a lighter color indicates lower content.

According to GC retention indexes (NIST 2020) and migration time databases in IMS, it was found that a total of 59 VOCs were detected in the five types of gardenia flower samples, mainly including 13 terpenes, 18 aldehydes, 4 esters, 8 ketones, 15 alcohols, and 1 sulfide. The qualitative results are shown in Table 1.

#### 2.2.3. GC–IMS Profile Analysis of VOCs in the Five Groups of Gardenia Flowers

**Figure 6:**
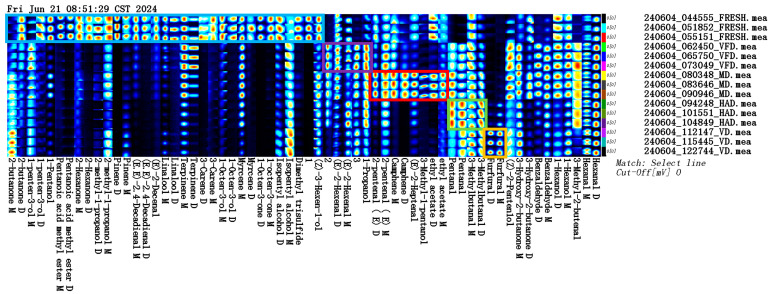
Fingerprint of VOCs in the five groups of gardenia flowers.

The construction of the fingerprint spectra of gardenia flowers using different drying methods can provide an effective means for evaluating gardenia flower quality. Figure 6 shows the fingerprint analysis of all the VOCs to further compare the sample VOCs. Each row represents all the selected signal peaks from a sample, and each column represents the same VOC in different samples. Brighter colors indicate higher content. By comparing and analyzing the volatile compounds in the fresh, VFD, MD, HAD, and VD samples, it was found that different drying methods significantly impacted the gardenia flower VOCs. The results show that the fresh group contained high levels of 2-butanone D, 1-penten-3-ol, 1-penten-3-ol D, 1-pentenol, pentanoic acid methyl ester D, pentanoic acid methyl ester M, 2-hexanone, 2-hexanone D, 2-hexanone M, 2-methylpropanol 2-methyl-1-propanol D, 2-methyl-1-propanol M, pinene D, pinene M, (E,E)-2,4-decadienal (E,E)-2,4-decadienal D, (E,E)-2,4-decadienal M, 2-decenal, (E)-2-decenal, linalool D, linalool M, terpinene M, 3-carene-M, 1-octen-3-ol, 1-octen-3-ol M, 1-octen-3-ol D, isopentyl alcohol D, dimethyl trisulfide, 3-hexenol, and (Z)-3-hexen-1-ol. As shown in the purple box, the 2-hexenal, (E)-2-hexenal D, and 1-propanol contents were higher under VFD. As shown in the red box, the 2-pentenal D, (E)-2-pentenal M, camphene D, camphene M, 2-heptenal, (E)-2-heptenal, (E)-3-methylpentenol, 3-methyl-1-pentanol, ethyl acetate M, and ethyl acetate D contents were higher under MD. As shown in the green box, the pentanal, 3-methylbutanal, 3-methylbutanal D, and 3-methylbutanal M contents were higher under HAD. As shown in the orange box, the furfural D and furfural M contents were higher under VD.

### 2.3. Chemometric Analysis

#### 2.3.1. Principal Component Analysis (PCA)

**Figure 7:**
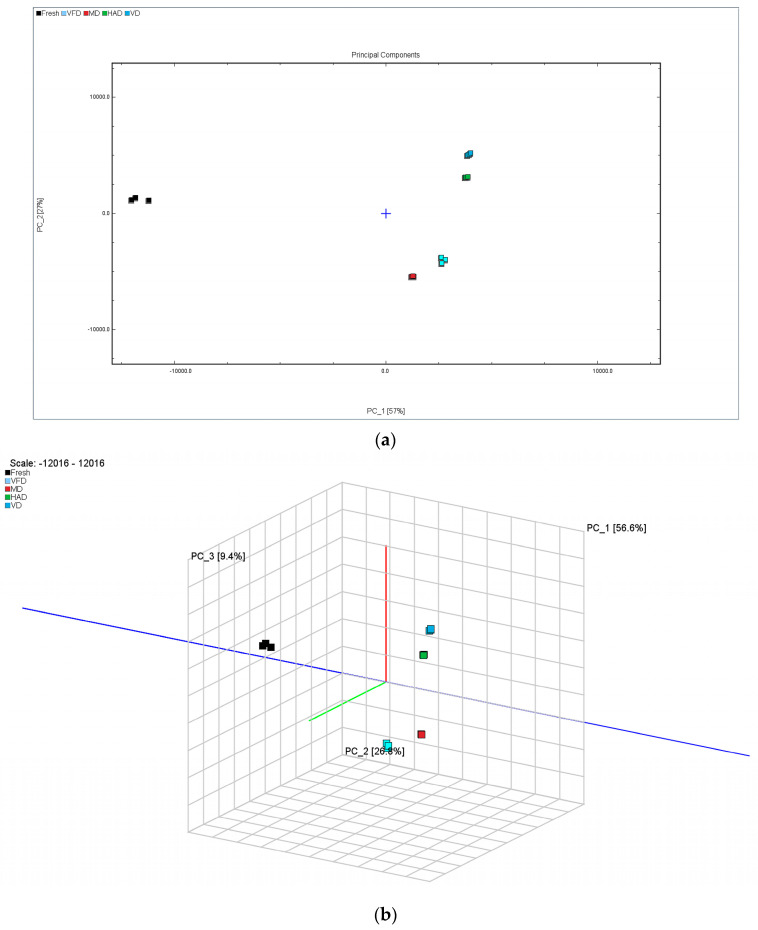
Principal component analysis (PCA) score plot of VOCs in the five groups of gardenia flower. (**a**) PCA score plot; (**b**) three-dimensional scatter plot.

Here, we utilized the Dynamic PCA plugin in VOCal data processing software to perform a PCA of the volatile organic compounds in the fresh and four different dried gardenia flower samples. Figure 7a shows that the first two principal components explained a cumulative contribution rate of 84% among the five sample groups, with PC1 contributing 57.0% and PC2 contributing 27.0%. The distance between HAD and VD, as well as that between VFD and MD, is relatively small, indicating small differences. However, there is a significant distance between the fresh group and the other four groups, indicating large differences and demonstrating that different drying methods have a significant impact on the VOCs of gardenia flowers. These differences can also be visualized in a three-dimensional scatter plot (Figure 7b). This PCA-based classification method was used to differentiate the VOCs of gardenia flowers dried under different methods, confirming that different drying methods have a significant impact on the VOCs of gardenia flowers.

#### 2.3.2. Cluster Analysis (CA)

**Figure 8:**
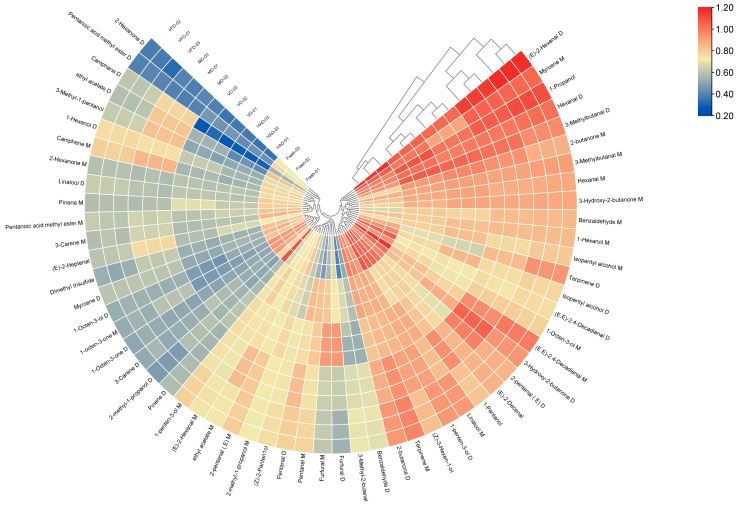
Cluster heatmap of VOCs in the five groups of gardenia flowers.

The 59 volatile component peaks from the fresh, VFD, MD, HAD, and VD groups were imported into TBtools software (v2.026) for cluster analysis. The heatmap visually represented the differences in the data between the different groups through changes in color intensity. It can be seen in Figure 8 that both the VFD and MD clusters and the HAD and VD clusters are clear, but the fresh group differs significantly from these. Compared to dried gardenia, the contents of 2-hexanone D, pentanoic acid methyl ester D, ethyl acetate D, 2-hexanone M, linalool D, pinene M, pentanoic acid methyl ester M, 3-carene M, dimethyl trisulfide, myrcene D, 1-octen-3-ol D, 1-octen-3-one M, 1-octen-3-one D, 2-methyl-1-propanol D, pinene D, 1-penten-3-ol D, linalool M, 1-pentanol, (E)-2-decenal, (E)-2-pentenal D, (E,E)-2,4-decadienal M, 1-octen-3-ol M, (E,E)-2,4-decadienal D, and isopentyl alcohol D were higher; the VFD group had a higher content of terpinene D; the MD group had higher levels of camphene D, ethyl acetate D, 3-methyl-1-pentanol, camphene M, (E)-2-heptenal, ethyl acetate M, and pentanal D; the VD group had higher levels of furfural M and furfural D; and the HAD group had higher levels of pentanal D and pentanal M. From the figure, it can be seen that there is a significant color difference between the HAD and VD groups compared to the fresh group, with some substances showing red and blue color blocks, representing a huge difference, at two extremes. In contrast, the VFD and MD groups show less change in the compounds compared to the fresh group, as the color change of the area where these two groups are located is similar to that of the fresh group, with only slightly darker or lighter colors and no significant changes.

#### 2.3.3. Partial Least-Squares Discriminant Analysis (PLS-DA)

**Figure 9:**
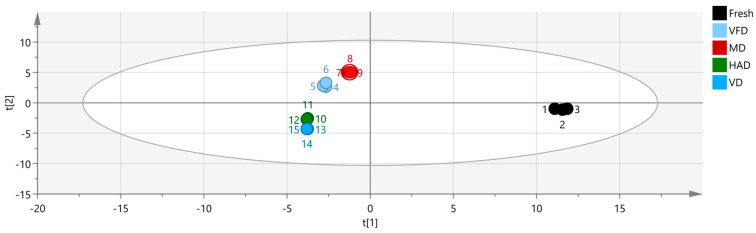
PLS-DA analysis of VOCs in the five groups of gardenia flowers.

**Figure 10:**
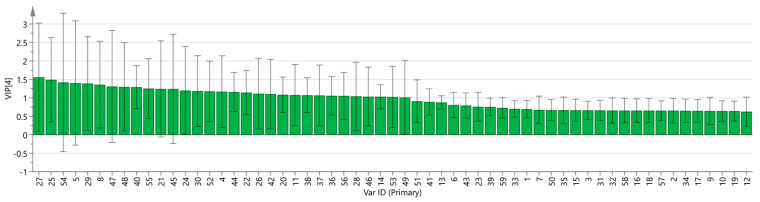
VIP values of the characteristic variables.

**Figure 11:**
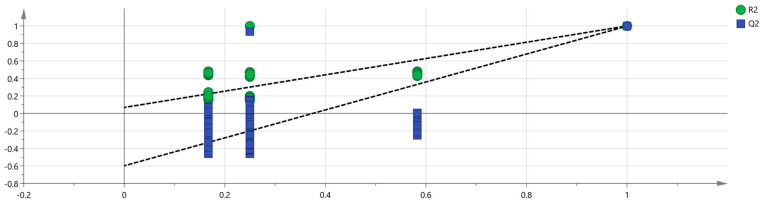
Permutation test results of VOCs in the five groups of gardenia flowers.

A PLS-DA model was developed to better observe the differences between the groups based on the different drying methods. PLS-DA is a supervised discriminant analysis method that differs from PCA, capable of interpreting observed values and predicting corresponding variables [24]. Using SIMCA software, the five sets of sample data were imported, and the results are shown in Figure 9. In the PLS-DA model, R^2^ and Q^2^ can evaluate model reliability and predictive ability. R^2^ and Q^2^ values greater than 0.5 indicate acceptable model fitting, while values close to 1 indicate strong predictive ability. The PLS-DA scoring chart shows that the model has good predictive ability (R^2^X = 0.995, R^2^Y = 0.997, Q^2^ = 0.998). The R^2^ and Q^2^ values are close to 1, indicating good fitting accuracy. The fresh group is distributed on the right side of the graph, while the other four groups of samples are all on the left side of the graph, indicating significant differences in the chemical composition of the fresh group compared to the other groups, consistent with the conclusion drawn from the PCA graph. In addition, a predicted variable importance projection (VIP) >1 is used as a standard to measure the strength and explanatory power of the expression pattern of compounds on the classification discrimination of each group of samples, thereby assisting in the screening of the main distinguishing peaks between the samples. As shown in Figure 10, 1-hexanol M, (E)-2-hexanal M, ethyl acetate M, terpinene D, furfural D, (E)-2-heptenal, 3-methyl-1-pentanol, (Z)-2-penten-1-ol isopentyl alcohol M, 1-propanol, camphene D, 3-methylbutanal D, (E)-2-hexanal D furfural M, 2-butanone M, terpinene M, pentanal D, hexanal D, (Z)-3-hexen-1-ol, 3-hydroxy-2-butanone D, benzaldehyde D, ethyl acetate D, and 1-penten-3-ol M are the main indicators of differences. These compounds are the main ones used to study the effects of the drying methods on the VOCs of gardenia flowers. At the same time, to determine whether the model is overfitted, we conducted 200 cross-validations to examine the R^2^ and Q^2^ values. From the graph, we observed that the slope of the line was large, indicating that the PLS-DA model was not overfitted (R^2^ = 0.0691, Q^2^ = −0.62; as shown in Figure 11).

## 3. Discussion

This study utilized GC–IMS technology to analyze the effects of different drying methods (vacuum freeze-drying, vacuum drying, microwave drying, hot-air drying) on the volatile compounds of gardenia flowers. A total of 59 volatile organic compounds were detected, i.e., 13 terpenes, 18 aldehydes, 4 esters, 8 ketones, 15 alcohols, and 1 sulfide. By using GC–IMS technology to obtain three-dimensional, two-dimensional, and color difference fingerprint spectra, we can clearly and intuitively observe significant differences in the volatile compounds of gardenia flowers dried under different methods. Meanwhile, the choice of drying method significantly impacts the appearance and color of dried gardenia flowers. Freeze vacuum-drying has the least impact on the appearance of gardenia flowers, with a color and appearance closest to those of fresh gardenia flowers; microwave-dried gardenia flowers exhibit slight browning and significant changes in appearance. In contrast, the samples dried by hot-air and vacuum drying showed a higher browning index, the darkest color, and the greatest changes in appearance.

In addition, according to the results of the volatile component fingerprint spectrum, it is known that the contents of 2-butanone D, 1-penten-3-ol D, 1-pentenol pentanoic acid methyl ester D, pentanoic acid methyl ester M, 2-hexanone D, 2-hexanone M, 2-methyl-1-propanol D, 2-methyl-1-propanol M, pinene D, pinene M, (E,E)-2,4-decadienal D, (E,E)-2,4-decadienal M, (E)-2-decanal linalool D, linalool M, terpinene M, 3-carene-M, 1-octen-3-ol M, 1-octen-3-ol D, isopentyl alcohol D, dimethyl trisulfide, and (Z)-3-hexen-1-ol are higher in the fresh group; (E)-2-hexenal D and 1-propanol have higher levels in the VFD group; (E)-2-pentenal D, (E)-2-pentenal M, camphene D, camphene M, (E)-2-heptenal, 3-methyl-1-pentanol, ethyl acetate M, and ethyl acetate D have higher levels in the MD group; pentanal, 3-methylbutanal D, and 3-methylbutanal M have higher levels in the HAD group; and furfural D and furfural M have higher contents in the VD group. Due to the varying levels of chemical components in gardenia flowers processed by different drying methods, the selection of drying methods should be consistent with the desired characteristics of the final product. Most of the monoterpenes and sesquiterpenes in fresh gardenia flowers have pharmacological activities of improving sleep, anti-anxiety, and anti-depression [24]; VFD promotes the synthesis of (E)-2-hexenal D with green, banana, and fat flavors. It is hoped that (E)-2-hexenal D can be recovered from gardenia waste as a food additive for food preservation [25]. At the same time, natural edible flavoring (E)-2-hexenal D is a potential antifungal agent [26,27]. Microwave-dried gardenia contains the highest amount of camphene, which has the function of lowering blood lipids [28]. After HAD, the gardenia flower has a high content of 3-methylbutanal, which has chocolate and fat flavors. 3-methylbutanal is a key flavor compound in many hard and semi-hard cheese varieties [29]. HAD gardenia flowers can be used as seasoning materials to add a fruit or cream aroma to food. HAD has a low cost, and samples can be processed in large quantities. The vacuum-dried gardenia flower has a high content of furfural, which is a dietary risk factor [30] and is classified as a Group 3 carcinogen on the 2017 International Agency for Research on Cancer List by the World Health Organization, posing a carcinogenic risk.

An analysis of the PCA results indicated that PCA1 and 2 accounted for 56.6 and 26.8%, respectively, of the cumulative contribution rate, amounting to 83.4% in total. This indicates that the two principal components can better reflect the information contained in the original data. The PCA results confirm that the differences between the VFD and MD samples and between the HAD and VD samples are relatively small, while the fresh samples differ distinctively from the other four groups of samples, indicating significant differences between them. In addition, based on the results of heat map clustering and PLS-DA analysis, we can clearly observe that VFD and MD and HAD and VD are closer to each other, while the fresh samples are significantly different, further verifying the conclusions obtained earlier. From the VIP results, it can be seen that different drying methods have the greatest impact on volatile substances, such as 1-hexanol M, (E)-2-hexanal M, and ethyl acetate M, in gardenia flower samples.

High temperature and dry methods accelerate the volatilization and degradation of VOCs in gardenia flowers, particularly those with high heat sensitivity, such as certain esters. High temperatures may also trigger chemical reactions, such as the Maillard reaction, further altering the composition of VOCs. A low temperature can better protect VOCs. Rapid freezing and low-temperature vacuum drying can maximize the retention of the original aroma components of gardenia flowers. The VOCs in gardenia flowers are often closely associated with water molecules. During the drying process, as water evaporates, VOCs are gradually released and may undergo changes. Different drying methods have varying impacts on the speed and manner of water evaporation, leading to changes in VOCs. Additionally, prolonged drying processes may result in continuous volatilization and degradation of VOCs in gardenia flowers, especially those that are unstable.

In summary, different drying methods influence VOCs in gardenia flowers through various factors, such as temperature, water status, drying time and efficiency, and other factors, leading to varying degrees of changes. In practical applications, the appropriate drying method should be selected based on specific needs and conditions to maximize the retention of aroma components and biological activity of gardenia flowers.

## 4. Materials and Methods

### 4.1. Materials

The gardenia flowers were provided by Hunan Hi-Tech Bio-Agro Co., Ltd., Yueyang, China. A voucher specimen (HNATCM2024-ZZH001) was stored in the sample room of the Science and Technology Innovation Center of Hunan University of Chinese Medicine, Changsha, China.

### 4.2. Drying Procedures

All the fresh gardenia flowers were dried to a constant weight. For VFD, 500 g of fresh gardenia flowers were pre-frozen at −80 °C and then dried by a freeze-dryer (Alpha 2-4 LSCbasic, CHRIST, Hagen, Germany) for 8 h; the temperature and pressure were −80 °C and −0.1 MPa, respectively. For HAD, 500 g of fresh gardenia flowers were maintained in a ZXRD-A7140 electric convection oven (Zhicheng Analytical Instrument Manufacturing Co., Ltd., Shanghai, China) for 6 h at 60 °C and an air velocity of 0.4 m/s. For VD, 500 g of fresh gardenia flowers were dried in a BZF-50 vacuum oven (Boxun Medical Biological Instrument Co., Ltd., Shanghai, China) at −0.09 MPa and 60 °C for 6 h. For MVD, 500 g of fresh gardenia flowers were placed in a microwave oven (WB-5, Famouk Co., Ltd., Fuzhou, China) and dried at 560 W for 1.5 h; the temperature was maintained at 60 °C and the pressure at −0.08 MPa. The dried gardenia flowers were then ground into powders until they were required for analysis.

### 4.3. Analysis by GC–IMS

The VOCs in the dried powders were analyzed directly by headspace-gas chromatography–ion mobility spectrometry (HS-GC–IMS) using a FlavorSpec^®^ Gas Phase Ion Mobility Spectrometer from GAS (Dortmund, Germany).

From each sample (1 g), the powder was placed in a 20 mL headspace vial and incubated for 20 min at 40 °C, after which 500 µL was injected into the headspace by non-shunt injection, and the vials were rotated at 500 rpm for 20 min (injection needle temperature: 85 °C). The VOCs were separated on an MXT-WAX capillary column (15 m × 0.53 mm × 1.0 mm, Restek Inc., Edmond, OK, USA) and maintained at 60 °C using high-purity N_2_ as the carrier gas. Initially, the flow rate was 2.00 mL/min; within 8 min, it was linearly increased to 10.00 mL/min, then to 100.00 mL/min within 10 min, and was finally held for 10 min. The chromatography runtime was 30 min, and the injection temperature was 85 °C. The IMS conditions were as follows: 3H (tritium) was used as the IMS reagent, a 53 mm drift tube was used, the electric field intensity was 500 V/cm, the drift tube temperature was 45 °C, high-purity N_2_ (99.999%) was used as the carrier gas, the flow rate was 75 mL/min, and a positive ion mode was used.

### 4.4. Statistical Analysis

Chemometrics is typically described as the discipline of extracting additional information from large datasets using mathematical and statistical methods. It is a key tool for revealing complex chemical matrix patterns [31]. In the field of GC–IMS data analysis, the three commonly used chemometric techniques fall into two categories: exploratory analysis and classification methods. Principal component analysis (PCA) is a technique for simplifying complex data by transforming many interrelated raw variables into several orthogonal principal component variables [32]. These components can be used to evaluate sample similarities and differences [33]. Furthermore, cluster analysis (CA) and partial least-squares discriminant analysis (PLS-DA), as representatives of classification methods, finely classify samples based on extracted features. This series of methods has been widely applied in multiple fields, including but not limited to component analysis of traditional Chinese medicine, the quality assessment of agricultural products, the differentiation of food types, and sample classification in medical diagnosis, greatly promoting the accuracy and efficiency of data analysis in these fields.

Three-dimensional, two-dimensional, differential, fingerprint, and principal component analysis (PCA) spectra of the VOCs were generated using plugins such as Reporter, Gallery Plot, and Dynamic PCA in VOCal data processing software (from G.A.S., Dortmund, Germany, version 2.0.0). CA and PLS-DA were conducted using TBtools and SIMCA (Version 14.1, Umetrics, Sweden), respectively.

## 5. Conclusions

This study analyzed and compared VOCs in fresh gardenia flower samples and those after four different drying methods using GC–IMS combined with chemometric techniques. The results indicate differences in the appearance and color of the gardenia flowers under different drying methods. Significant differences exist in the VOC types and contents between the fresh gardenia flowers and those treated with different drying methods.

Through data analysis including PCA, CA, and PLS-DA, the data were visualized, confirming the differences in the VOCs between fresh gardenia flowers and those treated with different drying methods. These research results will contribute to the precise development of volatile-component-related products of gardenia flower with corresponding functions using different drying methods.

Overall, VFD or MD may be the most effective alternative to traditional sun-drying methods. Considering its drying efficiency and production cost, MD has the widest market prospects.

This study utilized GC–IMS technology to determine the content of VOCs in gardenia flower samples treated with different drying methods. Combined with chemometrics, the differences in the VOCs of gardenia flowers can be visually discerned. This study will facilitate the comprehensive development and utilization of VOCs of gardenia in cosmetics, health products, functional foods, natural food seasonings, and preservatives.

## Figures and Tables

**Table 1:** Results of component analysis of VOCs in five groups of gardenia flowers.

No	Compounds	CAS#	Molecular Formula-	MW	RI	Rt/s	Dt/ms
1	(E,E)-2,4-Decadienal M	C25152845	C_10_H_16_O	152.2	1307.9	1446.213	1.38142
2	(E,E)-2,4-Decadienal D	C25152845	C_10_H_16_O	152.2	1309.0	1450.998	2.06720
3	(E)-2-Decenal	C3913813	C_10_H_18_O	154.3	1320.2	1500.447	1.46749
4	Terpinene M	C99854	C_10_H_16_	136.2	1059.1	690.082	1.20729
5	Terpinene D	C99854	C_10_H_16_	136.2	1060.2	692.312	1.71423
6	Linalool M	C78706	C_10_H_18_O	154.3	1100.9	781.484	1.21490
7	Linalool D	C78706	C_10_H_18_O	154.3	1101.9	783.713	1.74465
8	(E)-2-Heptenal	C18829555	C_7_H_12_O	112.2	960.6	499.773	1.25167
9	3-Carene D	C13466789	C_10_H_16_	136.2	1006.8	590.694	1.64996
10	3-Carene M	C13466789	C_10_H_16_	136.2	1006.5	590.103	1.24163
11	Myrcene M	C123353	C_10_H_16_	136.2	995.5	570.620	1.17301
12	Myrcene D	C123353	C_10_H_16_	136.2	992.7	564.716	1.69179
13	Benzaldehyde M	C100527	C_7_H_6_O	106.1	965.5	509.220	1.15293
14	Benzaldehyde D	C100527	C_7_H_6_O	106.1	964.6	507.448	1.46420
15	1-Octen-3-ol M	C3391864	C_8_H_16_O	128.2	986.9	552.318	1.15460
16	1-Octen-3-ol D	C3391864	C_8_H_16_O	128.2	988.6	555.861	1.59808
17	1-Octen-3-one M	C4312996	C_8_H_14_O	126.2	970.9	519.847	1.26505
18	1-Octen-3-one D	C4312996	C_8_H_14_O	126.2	970.6	519.256	1.67673
19	Dimethyl trisulfide	C3658808	C_2_H_6_S_3_	126.3	961.7	501.977	1.31297
20	Camphene M	C79925	C_10_H_16_	136.2	940.8	463.618	1.20876
21	Camphene D	C79925	C_10_H_16_	136.2	944.4	469.967	1.63800
22	Hexanal D	C66251	C_6_H_12_O	100.2	793.9	267.873	1.55140
23	Hexanal M	C66251	C_6_H_12_O	100.2	798.5	272.432	1.25581
24	(E)-2-Hexenal D	C6728263	C_6_H_10_O	98.1	861.3	343.782	1.51926
25	(E)-2-Hexenal M	C6728263	C_6_H_10_O	98.1	852.9	333.232	1.17816
26	(Z)-3-Hexen-1-ol	C928961	C_6_H_12_O	100.2	839.5	317.176	1.22790
27	1-Hexanol M	C111273	C_6_H_14_O	102.2	873.1	359.193	1.32284
28	1-Hexanol D	C111273	C_6_H_14_O	102.2	871.4	356.958	1.64838
29	Furfural D	C98011	C_5_H_4_O_2_	96.1	835.6	312.543	1.32538
30	Furfural M	C98011	C_5_H_4_O_2_	96.1	833.6	310.308	1.08091
31	Pentanoic acid methyl ester M	C624248	C_6_H_12_O_2_	116.2	820.9	296.062	1.22278
32	Pentanoic acid methyl ester D	C624248	C_6_H_12_O_2_	116.2	822.2	297.458	1.55971
33	1-Pentanol	C71410	C_5_H_12_O	88.1	774.5	248.573	1.52931
34	2-Hexanone D	C591786	C_6_H_12_O	100.2	786.8	260.865	1.49764
35	2-Hexanone M	C591786	C_6_H_12_O	100.2	786.2	260.306	1.19744
36	3-Methyl-2-butenal	C107868	C_5_H_8_O	84.1	788.5	262.541	1.35958
37	2-Pentenal (E) D	C1576870	C_5_H_8_O	84.1	753.4	228.461	1.35958
38	2-Pentenal (E) M	C1576870	C_5_H_8_O	84.1	757.4	232.092	1.10624
39	Isopentyl alcohol D	C123513	C_5_H_12_O	88.1	739.6	216.170	1.49511
40	Isopentyl alcohol M	C123513	C_5_H_12_O	88.1	737.0	213.935	1.23418
41	3-Hydroxy-2-butanone M	C513860	C_4_H_8_O_2_	88.1	719.1	199.130	1.06697
42	3-Hydroxy-2-butanone D	C513860	C_4_H_8_O_2_	88.1	718.0	198.292	1.32791
43	Pentanal M	C110623	C_5_H_10_O	86.1	706.1	189.074	1.19744
44	Pentanal D	C110623	C_5_H_10_O	86.1	705.4	188.515	1.41911
45	3-Methylbutanal D	C590863	C_5_H_10_O	86.1	671.6	166.726	1.40011
46	3-Methylbutanal M	C590863	C_5_H_10_O	86.1	669.0	165.330	1.18351
47	3-Methyl-1-pentanol	C589355	C_6_H_14_O	102.2	847.8	327.069	1.61165
48	(Z)-2-Penten1ol	C1576950	C_5_H_10_O	86.1	768.0	242.149	0.94284
49	1-Penten-3-ol M	C616251	C_5_H_10_O	86.1	691.7	178.459	0.94284
50	1-Penten-3-ol D	C616251	C_5_H_10_O	86.1	695.6	181.252	1.34818
51	2-Butanone D	C78933	C_4_H_8_O	72.1	614.2	138.249	1.24753
52	2-Butanone M	C78933	C_4_H_8_O	72.1	608.5	135.712	1.05940
53	Ethyl acetate D	C141786	C_4_H_8_O_2_	88.1	638.2	149.516	1.33044
54	Ethyl acetate M	C141786	C_4_H_8_O_2_	88.1	634.1	147.529	1.09712
55	1-Propanol	C71238	C_3_H_8_O	60.1	553.1	113.251	1.12415
56	2-Methyl-1-propanol M	C78831	C_4_H_10_O	74.1	647.2	153.987	1.17910
57	2-Methyl-1-propanol D	C78831	C_4_H_10_O	74.1	646.2	153.490	1.37188
58	Pinene D	C80568	C_10_H_16_	136.2	928.2	441.947	1.67817
59	Pinene M	C80568	C_10_H_16_	136.2	931.0	446.754	1.29509

## Data Availability

Data are contained within the article.
